# On the thermodynamic path enabling a room-temperature, laser-assisted graphite to nanodiamond transformation

**DOI:** 10.1038/srep35244

**Published:** 2016-10-12

**Authors:** F. Gorrini, M. Cazzanelli, N. Bazzanella, R. Edla, M. Gemmi, V. Cappello, J. David, C. Dorigoni, A. Bifone, A. Miotello

**Affiliations:** 1 Laboratorio IdEA, Dipartimento di Fisica, Università degli Studi di Trento, via Sommarive 14, I-38123 Povo (TN) Italy; 2 Istituto Italiano di Tecnologia, Center for Neuroscience and Cognitive Systems, corso Bettini 31 Rovereto (TN) Italy; 3 Istituto Italiano di Tecnologia, Center for Nanotechnology Innovation, piazza San Silvestro, 12 Pisa, Italy

## Abstract

Nanodiamonds are the subject of active research for their potential applications in nano-magnetometry, quantum optics, bioimaging and water cleaning processes. Here, we present a novel thermodynamic model that describes a graphite-liquid-diamond route for the synthesis of nanodiamonds. Its robustness is proved via the production of nanodiamonds powders at room-temperature and standard atmospheric pressure by pulsed laser ablation of pyrolytic graphite in water. The aqueous environment provides a confinement mechanism that promotes diamond nucleation and growth, and a biologically compatible medium for suspension of nanodiamonds. Moreover, we introduce a facile physico-chemical method that does not require harsh chemical or temperature conditions to remove the graphitic byproducts of the laser ablation process. A full characterization of the nanodiamonds by electron and Raman spectroscopies is reported. Our model is also corroborated by comparison with experimental data from the literature.

Nanodiamonds (NDs) present interesting properties, such as high hardness, thermal conductivity and resistance to friction^1^. Moreover, NDs can be implanted to obtain Nitrogen-Vacancy centers^2^,^3^ (NV-centers), whose spin-dependent fluorescence enables ultrasensitive detection of electric and magnetic fields at the nanoscale (nano-magnetometry^4^ and nano-electrometry^5^), with applications in nano-thermometry^6^ and as single photon emitters in quantum optics^7^. Due to their biocompatibility^8^ and to the possibility of surface functionalization, NDs have also been proposed as nanoprobes for biomedical applications, including imaging^9^ and drug delivery^10^.

Production of NDs is a challenge as it requires extreme pressure and temperature conditions. Several methods have been proposed, including detonation^11^, high-pressure-high-temperature (HPHT) growth^1^, ultrasound cavitation^12^, chemical vapor deposition^13^ and laser-assisted techniques^14^,^15^,^16^.

Among the methods relying on laser irradiation, confined-pulsed-laser-ablation (CPLA) has been proposed as a potentially scalable and cost effective process for the production of NDs. In this approach, laser nanosecond pulses were applied to graphite confined between solid, transparent substrates that helped create favorable conditions for the formation of NDs^17^. While potentially useful for a number of applications, including laser writing of patterned diamond films, harvesting of the NDs, e.g. for their use as nanoprobes in bioimaging applications, remains a problem.

Here we report on the production of micrometric-sized crystalline clusters of NDs by CPLA in a water environment at ambient conditions. Specifically, we perform laser ablation of a substrate of graphite under a water layer that serves both as a confinement factor and as a medium for the suspension of the ablation products. Moreover, we propose a safe method for the removal of the graphitic components of the ablation products. A relevant section of the paper is related to the thermodynamic model we proposed to explain the experimental results. Starting from established models^18^, we apply the theory of homogenous nucleation in an undercooled liquid to explain appearance and growth of NDs under the conditions used in our experiment.

## Results and Discussion

**Figure 1:**
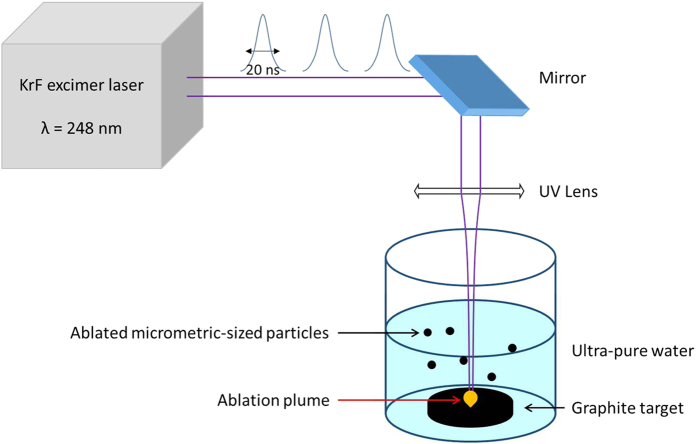
Setup of CPLA process. The UV laser output is focused through a quartz lens (focal length f = 40 cm) and irradiates the pyrolytic graphite target. Ablated particles are suspended in water.

Our CPLA experiment is presented in Fig. 1. We focused a controlled number of KrF excimer high-power laser shots on a graphite target in a water-confined geometry. Water itself acts as the confinement medium, where the ablated graphite particles as well as nano- and micro-clusters of assembled NDs are dispersed.

**Figure 2:**
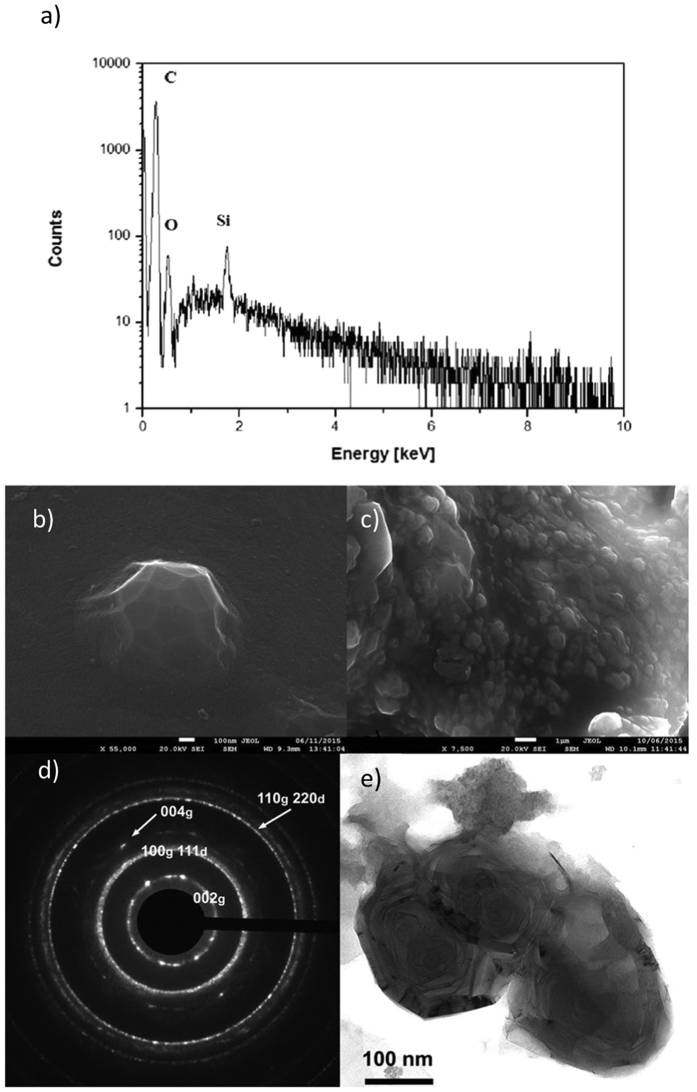
Experimental analysis of typical crystallites of intergrown NDs and graphite. Panel (a) shows EDS spectrum taken on crystallites as in panels (b,c). The crystallites are composed by carbon and the peak of silicon comes from the substrate, where the sample powders were deposited. Oxygen is always present as a superficial contamination of crystallites. Panel (b) shows a single crystallite with linear size of about 800 nm and panel (c) typical agglomerate of crystallites of 500 nm average size. Panel (d) displays the electron diffraction collected on the particles shown in panel (e). The diffraction rings are indexed accordingly to the graphite (subscripts g) and the diamond structure (subscript d). Panel (e) Bright field TEM image of two graphitic-embedded crystallites.

**Figure 3:**
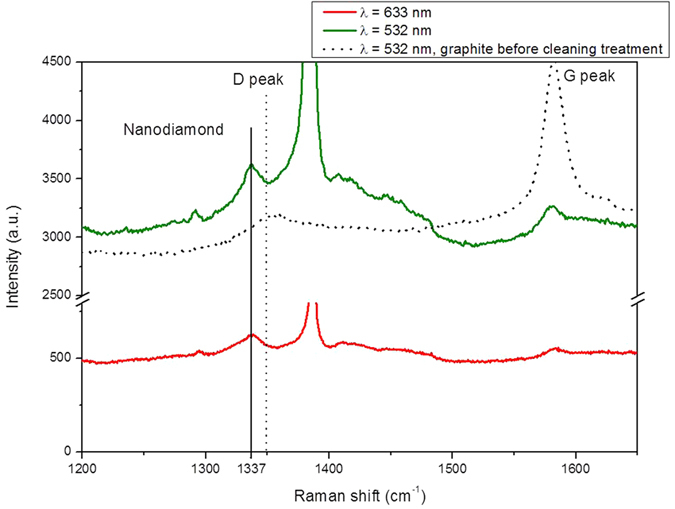
Raman spectroscopic analyses. Raman spectra of chemically treated NDs taken at two different excitation wavelengths (green and red line), and of untreated ablated graphitic powder (dotted line). A peak at 1337 cm^−1^ becomes clearly visible after the UV/chemical cleaning. After chemical treatment, graphite is still present with a peak at 1580 cm^−1^ (“G peak”), while the graphitic “D peak” at 1350 cm^−1^ is not detected. The peak at 1380 cm^−1^ comes from contaminants and was attributed to sodium nitrate present on the graphite and nanodiamonds’ surface.

The ablated carbon materials were collected in the water itself and treated with UV light in H_2_O_2_ environment (see Methods) in order to separate as much as possible the graphitic phase from the NDs. SEM images (Fig. 2) clearly indicate the presence of single and clustered crystallites attributed to NDs agglomerates having a typical size in the range 0.1–1 μm. Both isolated and clustered crystallites were surrounded by a graphitic matrix. Energy dispersive spectroscopy (EDS) (Fig. 2a) shows that the crystals are composed almost exclusively of carbon. An electron diffraction pattern –Fig. 2d)- taken on a graphitic-embedded crystallite –Fig. 2e)- exhibits reflection rings that can be indexed as (002 n)_g_ reflections of a graphite structure with an interlayer spacing of 3.45 Å characteristic of a disordered stacking of graphitic layers, plus reflections that are compatible with a diamond structure. Among these a large ring which can contain at the same time the 2.13 Å periodicity ((100)_g_ of graphite) and the 2.05 Å periodicity of (111)_d_ of diamond and a well defined ring of periodicity 1.26 Å which can be indexed at the same time as (110)_g_ and (220)_d_. In order to evaluate the relative content of the carbon hybridizations, *sp*^2^ vs *sp*^3^, we analyzed the ablation products with Raman spectroscopy at 633 nm and 532 nm laser excitation wavelengths. Carbon with *sp*^2^-hybridized bonds undergoes a double-resonant Raman scattering^19^ that dominates the Raman spectrum, independently of the excitation wavelength (Fig. 3). Ablated material displayed both the G-peak and the D-peak, indicating the appearance of disorder in the form of carbon nanoparticles. Partial removal of the graphitic component by UV treatment in H_2_O_2_ (see Methods) enabled detection of the characteristic Raman peak of diamond at around 1332 cm^−1^. Our samples showed a peak at 1337 cm^−1^ that is commonly attributed to compressively-strained nano-crystalline diamonds^20^,^21^. Compressive strain blue-shifts the line of diamond^22^ from its typical position at 1332 cm^−1^ and causes the broadening^23^ from ≈4 cm^−1^ to ≈10 cm^−1^. The graphitic G-peak is still visible, while the D-peak is not detected. The difference in the heights of the green and red spectra is due to the higher intensity of the green line compared to the red. The peak at 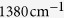 could be due to contaminating sodium nitrate^24^ from water and was collected from the micrometric-sized laser excitation region of the Raman experiment.

**Figure 4:**
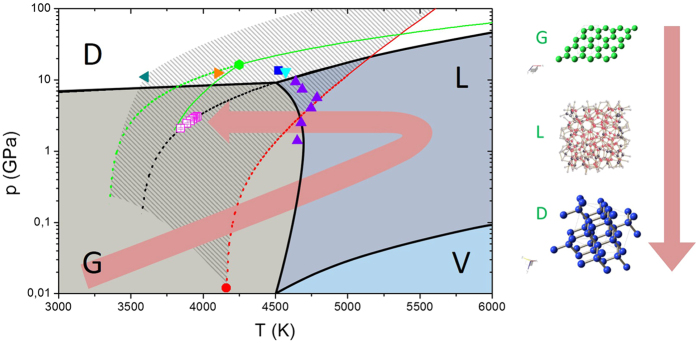
The phase diagram of carbon. The four regions of diamond D (white), graphite G (light grey), gaseous carbon V (light blue) and liquid carbon L (blue) are separated by coexistence lines (in black^44^). The coexistence line between diamond and liquid carbon (DL-line) falls in the grey-dashed region, which is bounded by the green DL-line (Ghiringhelli *et al*.^42^), and by the red DL-line (as described by Basharin^39^), and encloses the black DL-line (according to Zazula^41^). The dashed lines indicate the analytical continuations of the three curves. The dashed lines define a stable or metastable region for diamond nucleation. The red dot represents a metastable diamond at p = 0.012 GPa and T = 4160 K, obtained by quenching liquid carbon^39^. The purple triangles indicate the region of graphite melting measured by Togaya^45^. The dark green leftward triangle, orange rightward triangle, light green hexagon, blue square and light blue downward triangle represent the diamond-graphite-liquid triple points (DGL-points) according to Fateeva^46^, Bundy^44^, Ghiringhelli^42^, Glosli et Ree^47^, and van Thiel et Ree^48^, respectively. The purple squares describe our experimental system crossing the DL-line of Zazula from the right and becoming an undercooled liquid. Pressures of the purple squares were calculated from experimental laser intensities^29^. The red arrow indicates the evolution of the system in our experiment: from ambient temperature and pressure to liquid carbon, then to an undercooled liquid and finally to metastable diamond and graphite.

In order to explain the formation of NDs (Figs 2 and 3) from graphite under laser irradiation treatment and water confinement, we have modeled the path in the phase diagram under the thermodynamic constraints that govern this transition (Fig. 4).

The confining medium was water in the present case (confining liquids are reported in many articles^14^,^25^,^26^,^27^,^28^), while confining solid, glass/quartz, are reported by Qiong Nian *et al*.^15^.

Regardless of the type of confinement, the CPLA that permits the diamond phase formation can be described as a three-step process:

1. First, during high power laser irradiation, a very intense plasma plume is created at the graphite-water interface and pressure and temperature reach their peak values of 1–10 GPa and 5000–6000 K, respectively^29^,^30^,^31^,^32^. These values fall in the liquid region of the carbon phase diagram describing the appearance of a melt layer between the plume and graphite of the bulk target.
2. Upon expansion of the plume, temperature and pressure decrease. Heat is also conveyed to water and, if the cooling rate is sufficiently high, the liquid carbon becomes super cooled, and at this point nucleation of NDs is initiated. Liquid droplets formation, as quantitatively described in the paper of Mazzi *et al*.^33^ helps to explain the formation of NDs.
3. Growth of diamond nuclei continues as long as pressure and temperature favor the crystallization of liquid into diamond nuclei.

The transformation process is thus in the form graphite-liquid carbon-diamond (compare to the work of X.D. Ren *et al*.^17^). The presence of a liquid phase is essential, since the model considers the nucleation and the growth of diamond crystals in an undercooled liquid.

Description of the interaction of the high-power laser pulse with the graphite layer by heat transport equation and related boundary conditions requires knowledge of several thermodynamic and optical parameters that are not experimentally available. To avoid use of several free parameters to calculate pressure-temperature values induced in the irradiated graphite sample, we made use of a simpler model, known as Fabbro’s model. This model is generally accepted for the estimation of pressure in the plume, which represents the recoil pressure acting on the graphite target^29^. Fabbro’s model is rigorously derived from the first principle of thermodynamics, under the hypothesis of ideal gas behavior of the vapor plume, and states that the evolution of pressure at the graphite-target interface depends on the intensity of the laser *I.* A simple solution considers constant laser intensity *I*_0_ during the laser pulse-length τ. Then, pressure is also constant and given by:


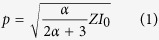


where *α* is the fraction of internal energy of plasma related to thermal energy, and 1 − α the ionization fraction of internal energy (usually *α* = 0.1 − 0.5). The quantities 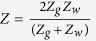 and *Z*_g_, *Z*_*w*_ are the reduced shock impedances of the system and the shock impedances of graphite and water, respectively. A number of papers^29^,^30^, mostly involving foils of Al, Cu, Zn and steel immersed in water, have shown a good agreement of this model with experimental data. In particular, during pulsed laser ablation of metal targets in water, the generated plume can easily attain pressures of 1–10 GPa for laser intensities of 1–10 GW/cm^2^. Berthe^30^ showed that, the absorption of confined plasma can be of 80–90% of the incoming energy, depending on laser pulse duration. Hence, *I*_0_ must decrease to 10–20% of the initial value when considering interaction with the graphite surface, in addition to the decrease provided by absorption by water. In order to calculate the pressure in eq. 1, we chose *α* ≈ 0.25 as in the paper of G.W. Yang^34^. The shock impedances^34^,^35^ were *Z*_*w*_ ≈ 1.65 × 10^6^ kg m^−2^ s^−1^ and 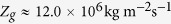. Therefore, 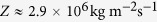. In the present work, the highest value of the effective intensity of the laser was 4.5 GW/cm^2^, corresponding to a pressure of 3.17 GPa. Temperature was estimated to be in the interval of 4500–6000 K, as in other works^31^,^32^. Actually, these conditions of temperature and pressures fall in the liquid region of the phase diagram of carbon (Fig. 4). The pressure exerted by the plasma/plume is so high that, in the inner layers, the transition occurs from graphite to liquid carbon. There are experimental evidences^36^,^37^ of graphite melting for laser fluences exceeding the threshold of 0.6 J/cm^2^, with a ruby laser (*λ* = 694.3 nm) and 30 ns of pulse duration. The melt depth increases almost linearly with the laser fluence and reaches a value of about 200 nm for 2−3 J/cm^2^. To our knowledge there are no available experimental data for our laser fluences, in the proximity of 100 J/cm^2^. However, analytical models that consider both vaporization and melting^38^ predict up to 1 μm thick melt layer at intensities of 10^13^ W/m^2^, as in our case. A thickness of several hundred nanometers is compatible with the size of our final structures (Fig. 2).

After melting, the system undergoes cooling, because the melt graphite is in thermal contact with the bulk graphite and surrounding water, and temperature and pressure decrease abruptly. Cooling velocity of the melted graphite is expected to be as high as 10^10^–10^11^ K/s (as usual with nanoseconds laser pulses) and, in this conditions, within a few nanoseconds and in undercooling state, the system is brought into the region of stability of diamond or metastability of graphite, where the nucleation of a solid stable phase (diamond) is favored^39^,^40^.

To estimate the size of the NDs obtained under our experimental conditions, we looked at the theory of the homogeneous nucleation where the critical radius r* (the radius of a nucleus at thermodynamic equilibrium with the surrounding liquid) is given by


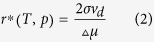


where *σ* is the surface tension, Δ*μ* is the difference between the chemical potential of diamond *μ*_d_ and the liquid carbon *μ*_*l*_ and *v*_*d*_ is the molecular volume of diamond.

The melting temperature *T*_*m*_(*p*), depending on the pressure, is located on the binodal line, defined by the Clausius-Clapeyron:


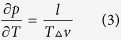


with Δ*v* = *v*_*l*_ − *v*_*d*_, *v*_*l*_ being the molecular volume of liquid carbon and *l* = 125 KJ/mol the enthalpy of fusion^41^. We refer to the work of Ghiringhelli *et al*.^42^ for a consistent evaluation of the set of quantities *T*_m_(*p*), *v*_d_ and *v*_l_. Surface tension is a function of both temperature and radius of NDs^43^:


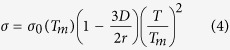


with *D *= 0.154 nm being the interatomic spacing of diamond and 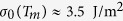.

The final size of the crystals was mainly due to the growth of diamond nuclei in the liquid. The growth velocity is expressed by


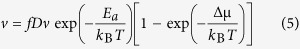


where *f* is the fraction of sites available for attachment (supposed to be ≈1), *E_a_* ≈ 250 kJ/mol is the molar adsorption energy necessary to cross the liquid-solid interface and *v* = 2.5·10^13^ Hz is the thermal vibration frequency^49^,^50^,^51^. To calculate the final diameter of NDs we made use of the formula 
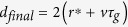
 where *τ*_g_ is the time available for crystal growth. In many cases this time is of the order of magnitude of the duration of the laser pulse^52^,^53^, so we set *τ*_g_ to twice the pulse-length of the laser.

**Figure 5:**
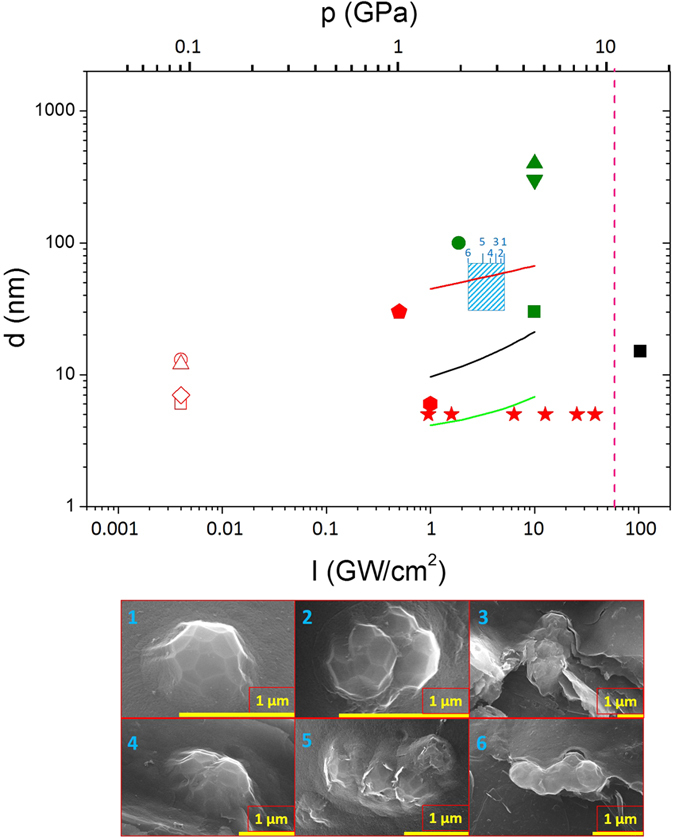
Experimental size of NDs as a function of laser intensity. The dashed-blue box represents the region in which the size of our single NDs crystals falls. It spans a range from 30 to 70 nm, centered around 50 nm, with laser intensities from 2.4 and 5.2 GW/cm^2^. Red points are taken from Sun^56^ (empty square), Hu^57^ (empty circle), Bai^58^ (empty diamond and empty triangle), Ferrari^19^ and Ren^59^ (hexagon and stars) and Mortazavi^54^ (pentagon) and correspond to ultrafine NDs produced with a 1064 nm laser. The black square was obtained with a 355 nm laser^32^. Finally, the green points correspond to diamond produced with a 532 nm, taken from^28^,^29^,^55^ (circle, upward triangle and downward triangle) and Wang^25^ (square). Solid points correspond to ns laser pulses (5–20 ns pulse lengths), while empty points correspond to laser pulses of 0.4–1.2 ms. The pink dotted line indicates the threshold for the region of absolute stability of diamond at 4500 K, i.e. the temperature of the DGL triple point according to Bundy. The red, black and green curves represent the theoretical size of a single diamond crystal as a function of pressure for a supercooled liquid crossing the DL-lines continuations of Basharin^39^, Zazula^41^ and Ghiringhelli^42^, respectively (same colors of Fig. 4). The apparent discrepancy between theory and data may be explained by the fact that the biggest crystals experimentally observed consist of agglomerates of smaller crystals (polycrystals). The six numbered SEM images indicate that size of the clusters do not vary appreciably with our laser intensities. The yellow bar in the bottom panels is always 1 μm long.

In Fig. 5, the blue-dashed box represents the region in which the size of our single NDs crystals falls. These crystallites are intermixed clusters of NDs and graphite, while the size of single NDs crystallites spans the range of 30–70 nm and doesn’t display great differences due to the change in laser intensity, from 2.4 and 5.2 GW/cm^2^. The theoretical estimations are in reasonable agreement with the experimental data, despite the fact that the present model considers only the thermodynamic state of the system and does not take into account laser wavelength and pulse duration, which, however, play a role mainly in the thermal history of the irradiated material.

In order to try to understand the role of the laser parameters, especially the wavelength, in determining the final NDs size, we compared our model with experimental data from the literature (graph of Fig. 5). The colors indicate the wavelength of the exciting laser: 1064 nm for the red points, 532 nm for the green points, 355 nm for the light blue point and 248 for the blue-dashed region. We also plot three curves showing the final size single-crystal, *d*_*final*_, due to nucleation and growth in a supercooled liquid, by considering the DL-lines of Basharin^39^, Zazula^41^ and Ghiringhelli^42^. The DL binodal line has still some uncertainty. In fact, for the laser intensity of 4.5 GW/cm^2^ the final size of NDs is found to be of 5.5 nm, 15.8 nm or 59.2 nm whether we account for the DL binodals of Ghiringhelli, Zazula or Basharin, respectively.

Apart from the pentagon^54^, NDs produced with IR laser are one order of magnitude smaller than NDs produced with green and UV lasers, regardless the intensity of the laser. A higher conversion of sp^2^ hybridized bonds to sp^3^ bonds has been reported for 193 nm laser compared to 1064 nm laser, with a bigger size of final NDs. Those authors (Mortazavi *et al*.^54^) argued that with IR laser there is a higher dissociation of the confining liquid molecules, thus causing strong interaction of C atoms with other non-carbon ionic species and the generation of different bonds additional to *sp*^3^ bonds. The size of our single crystals seems to confirm this trend. Further, the biggest structures (green points) are presumably intergrown polycrystals of cubic and hexagonal diamonds, according to authors^55^.

It must also be noticed that pulse duration seems to play a role in the phase transition process. Some of the crystals are obtained by laser pulses of 0.4–1.2 ms (empty points in Fig. 5). Clearly, in this case, the growth mechanism is different from nucleation in a super cooled liquid. Long heating time at relatively low intensity could allow the entire system to thermalize at the same temperature, with much lower cooling velocity at the end of the laser pulse.

Pressures calculated from the intensity of the laser through Eq. 1 in our experimental conditions or in literature reports, are lower than the pressures corresponding to the region of diamond phase (Figs 4 and 5). In some cases^56^,^57^,^58^, the estimated pressure is about 0.1 GPa, much less than the about10 GPa of phase diagram in Fig. 4. Nonetheless, diamonds of various sizes were nucleated under these experimental conditions. This apparent inconsistency might be explained on the basis that the Fabbro’s model underestimates the pressures generated in the plume acting on liquid carbon, in the case of graphite. This is not surprising, because of the complexity of the problem of the laser-surface interaction in high energy-density regime.

## Conclusion

We have demonstrated that CPLA of graphite in water under ambient conditions is a viable route to produce NDs suspended in an aqueous medium. Moreover, we proposed a safe procedure to extract the NDs from the embedding graphite. The NDs produced with our method can be delivered in a water suspension, a formulation that is advantageous for biological applications, e.g. for administration to cell and tissue cultures. A relevant section of the paper is related to a new thermodynamic model we have developed to explain the formation and growth of NDs under the experimental conditions used in the present study. Finally, several experimental results taken from literature have been considered/analyzed, to be possibly included in the present thermodynamic framework.

## Methods

In our CPLA experiment, a target of graphite was ablated by a KrF excimer laser (Coherent LPX220i) with *λ* = 248 nm, pulse-length *τ* = 20 ns, repetition rate of 10 Hz and 9000–12000 laser shots. We chose a high number of pulses to increase the ablated material dispersed in water. The energy of the laser was varied from ≈250 mJ to ≈600 mJ, and the beam was focused to fractions of mm^2^, achieving laser intensities in the range of 1–10 GW/cm^2^. The graphite target (a disk obtained by cutting a pyrolytic graphite rod) was covered by few mm’s of water during the irradiation and, if necessary, water was added to restore the initial level. The high energy deposited on the graphite target in the water medium created dispersed graphite particles alongside with embedded NDs. After ablation, the graphite powders containing NDs were sonicated in acetone for 3 h and then irradiated with UV (365 nm) light in a solution of concentrated hydrogen peroxide for 6 h. The UV combined peroxide chemical treatment removed some of the graphite and exposed free ND crystals. It should be noticed that this procedure is substantially safer and simpler than other methods to remove graphitic residues from NDs^60^, typically involving boiling acids (perchloric, sulphoric, nitric, etc) or potentially explosive compounds (ammonium nitrate). The UV light speeds up the dissociation of hydrogen peroxide into the strong oxidizing hydroxyl radicals 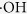.

For the characterization of the product, the samples were prepared by dispersing the graphite particles embedding the NDs into ultra-pure water. A solution droplet was then deposited on 1 × 1 cm^2^ Si wafer piece, and then soft backing was applied for 3–9 h to remove the water.

Morphological and compositional analyses were performed using a JEOL JSM-7001F Field Emission SEM equipped with an Oxford INCA PentaFETX3 EDXS detector. SEM-EDS analyses were performed at 20 keV energy beam and 10 mm of WD to allow simultaneous acquisition of morphological images and compositional spectra.

Electron diffraction and TEM imaging has been carried out on a Zeiss Libra 120 transmission electron microscope working at 120 kV and equipped with an in-column omega filter for energy filtered imaging. The sample was prepared by dispersion of the synthetized powder on isopropyl alcohol. A drop of the dispersion was then put on a carbon coated copper grid and let to dry out before observation.

A LabRam Aramis confocal microRaman system of Jobin-Yvon Horiba was used for the spectroscopic characterization of the same powders deposited on Si wafer. The two lines were provided by a He-Ne laser source (632.8 nm) and a DPSS laser source (532 nm). The signal was collected on an air-cooled multichannel CCD, with a wavenumber accuracy of ±1 cm^−1^ in the range between 450 nm and 850 nm.

## Additional Information

**How to cite this article**: Gorrini, F. *et al*. On the thermodynamic path enabling a room-temperature, laser-assisted graphite to nanodiamond transformation. *Sci. Rep.* **6**, 35244; doi: 10.1038/srep35244 (2016).
